# Electronic health records reveals resilience patterns of cardiovascular disease in Basque centenarians

**DOI:** 10.3389/fragi.2026.1805053

**Published:** 2026-03-31

**Authors:** Sara Cruces-Salguero, Igor Larrañaga, Javier Mar, Ander Matheu

**Affiliations:** 1 Cellular Oncology group, Biogipuzkoa (Biodonostia) Health Research Institute, San Sebastian, Spain; 2 Kronikgune Institute for Health Services Research, Barakaldo, Spain; 3 Biogipuzkoa Health Research Institute, San Sebastian, Spain; 4 IKERBASQUE, Basque Foundation for Science, Bilbao, Spain; 5 Centro de Investigación Biomédica en Red de Fragilidad y Envejecimiento (CIBERfes), Carlos III Institute, Madrid, Spain

**Keywords:** biology, cardiovascular, electronic health records, EHR, resilience, survival

## Abstract

**Background:**

Cardiovascular diseases (CVDs) are the leading cause of death worldwide. Although their prevalence increases with age, their impact on centenarians remains poorly understood.

**Methods:**

We analyzed Electronic Health Records (EHRs) to assess the incidence and effect of CVDs in centenarians (n = 649) and non-centenarians (n = 62,753) in the Basque Country. Descriptive statistics were applied to discern differences between the two population groups in terms of prevalence, number of diagnoses, and treatments. Survival analysis was performed through Kaplan-Meier estimator. Trajectories of laboratory parameters were explored through non-linear mixed models (NLMMs).

**Results:**

Centenarians had lower incidence of severe conditions such as ischemic heart disease, despite overall CVD incidence being similar between groups. Survival analyses revealed extended longevity in centenarians after both first and last CVD diagnosis. Biomarker trajectories suggested favorable biological profiles with greater resilience and faster recovery in centenarians.

**Conclusion:**

Despite experiencing CVDs, centenarians demonstrate delayed onset, reduced severity, and more favorable biological recovery compared to non-centenarians. These findings indicate potential resilience mechanisms that may contribute to healthy ageing.

## Introduction

Cardiovascular diseases (CVDs) constitute the leading cause of death globally, accounting for approximately 17.9 million deaths each year (World Health Organization, 2025a), which represents around of 32% of global deaths (World Health Organization, 2025b). These pathologies, which include ischemic heart disease, stroke, heart failure, and peripheral arterial disease, among others, are mainly caused by preventable environmental and behavioral risk factors such as physical inactivity, smoking, unhealthy diet and obesity (World Health Organization, 2025b).

Age is a key factor in the progressive disfunction of the cardiovascular system (North and Sinclair, 2012; Curtis et al., 2018), and the prevalence of CVDs increases with age (Yazdanyar and Newman, 2009; Rodgers et al., 2019). Indeed, the American Heart Association (AHA) reported that the incidence of CVDs increased from 40% in people aged 40–59 years until 86% in octogenarians (Yazdanyar and Newman, 2009). As world population is aging and it is anticipated an increasing number of older individuals in the future, the incidence and burden of CVDs will significantly rise, challenging the current healthcare systems (World Health Organization, 2025a). The onset and progression of CVDs in the old population has been a focus on research over last years, but still few studies have explored their prevalence and impact in the centenarian population.

Overall, current evidence on centenarians points towards a unique cardiovascular profile, with a delay in the onset and progression of disease (Galioto et al., 2008; Summer et al., 2025). However, there is controversy around the incidence of different CVDs in centenarians, with some studies reporting lower incidences of hypertension and myocardial infarction (Galioto et al., 2008), and others suggesting higher numbers of hypertension in centenarians (Zyczkowska et al., 2006). Moreover, in the Danish Longitudinal Centenarian Study, more than 72% of centenarians presented some form of CVD, such as hypertension (52%) and chronic heart failure (32%) (And et al., 2006). Additionally, studies on the centenarians’ offspring showed a reduced prevalence of age-related diseases, including cardiovascular conditions (Terry et al., 2003). Nonetheless, beyond incidence data, the way centenarians respond to CVDs, both clinically and biologically, remains poorly explored.

The Basque Country, located in the north of Spain, is one of the regions with the highest life expectancy in Europe, with 87 years for women and 82 years for men in 2023 (DatosMacro, 2025). Thus, it presents an important setting to study the centenarian population. Previous studies from the group characterized the centenarian population presenting fewer diseases, reduced use of healthcare services, and resilience to COVID-19 (Cruces-Salguero et al., 2022; Cruces-Salguero et al., 2024; Cruces-Salguero et al., 2025a; Cruces-Salguero et al., 2025b). Moreover, they presented unique molecular pathways and displayed better biological profiles at the end of their lives (Cruces-Salguero et al., 2022; Saenz-Antonanzas et al., 2024). Given the significant burden of CVDs, exploring the specific response of centenarians to this group of diseases could provide hints about their extended longevity and their potential resilience mechanisms against diseases and disability.

## Materials and methods

### Study population and design

An observational study explored cardiovascular diseases among centenarians retrospectively analyzing real-world data. We studied the population aged 48 years or older who were deceased between the 1^st^ of January of 2014 and the 31st of December of 2022, recorded in the database of Osakidetza (Basque Health Service).

First, the quality of data was assessed, and we excluded patients who had a date of birth and/or death without a valid format (dd/mm/yyyy) or with missing values, as well as patients with more than one dates of decease or with date of decease posterior to date of birth.

After data curation, 63,402 individuals remained. We explored demographic data, diagnoses records, pharmaceutic prescription, and laboratory analysis. For each patient, age was computed as the difference between the date of death and the date of birth. Non-centenarians were defined as individuals who were younger than 100 years at the time of decease (62,723 individuals, 98.93%), while centenarians were defined as individuals who were 100 years or older (679 individuals, 1.07%).

Diagnoses were recorded using diagnosis codes of the International Classification of Diseases, Ninth Revision (ICD-9), and included all diagnoses performed in primary care, outpatient, in-hospital care, and emergency. We selected diagnoses of circulatory system diseases that caused death of more than 1% of the population older than 50 years (Collaborators, 2018), as in previous publications of the group (Cruces-Salguero et al., 2025b). These were identified and classified through regular expressions based on ICD-9 codes. Related conditions such as diabetes and obesity were also considered. Diagnoses posterior to patients’ date of death were discarded. The list of the diagnoses is presented in Supplementary Table S1.

In the case of drugs, we first selected those belonging to the category ‘Cardiovascular system’ and then we also focused on ‘Antihypertensives’, lipid lowering drugs, and anti-diabetes medication, based on the Anatomical Therapeutic Chemical (ATC) code. Included codes were presented in Supplementary Table S1 **.**

The laboratory analysis included high-density lipoproteins (HDL), low-density lipoproteins (LDL), total cholesterol, creatinine, glucose, hemoglobin, glycosylated hemoglobin, International Normalized Ratio (INR), platelets, potassium, sodium, triglycerides, and urea. Outliers were filtered when it proceeded, based on the normal ranges of values for each variable. We included all available records for each patient, along with the date in which they were performed.

### Statistical analyses

We analyzed the incidence, numbers and proportions of records of CVDs and drugs in centenarians and non-centenarians through descriptive statistics. Student’s t-test or Mann-Whitney U test was applied depending on normality, assessed through the Shapiro-Wilk test. Survival of both groups since the first and last diagnosis of CVDs was assessed using the Kaplan-Meier estimator, as well as recurrence (second diagnosis of a CVD). Survival time was measured in years. Cox regression including group (centenarian or non-centenarian), age of first diagnosis (centered at the mean), sex, presence of diabetes and obesity, and prescription of CVD drugs was also built as a multivariate model for assessing survival since first CVD diagnosis. Differences in the last laboratory record between centenarians and non-centenarians were assessed using Student’s t-test or Mann-Whitney U test when it fitted, and multiple comparisons were corrected by false discovery rate (FDR). Trajectories of laboratory parameters were analyzed using non-linear mixed models (NLMMs) with four splines. For each patient, the year of the first CVD diagnosis was taken as the reference time point, and the subsequent evolution of each biological variable was examined according to whether the individual belonged to the centenarian or non-centenarian group. A separate model was built for each biological parameter, including the interaction between group and years relative to the first CVD diagnosis.

The construction and transformation of the database were performed using Python 3.9. All the statistical analyses were performed using R in RStudio software, v.4.3.3 (URL: https://www.r-project.org/). *Lme4* (Bates et al., 2015) package was used for NLMM, and s*urvival* (Therneau and Lumley, 2023) and *survminer* were used for the survival analysis.

## Ethics

This study was approved by the Basque Clinical Research Ethics Committee (CEIm-E code PI2020206), and adhered to the tenets of the Declaration of Helsinki of the World Medical Association on Human Experimentation. All the data was pseudo-anonymized, the authors did not receive information subject to re-identification, and procedures in order to protect pseudo-anonymization were performed by the data providers. The patients and the public were not involved in the design, or conduct, or reporting, or dissemination plans of our research.

## Results

**FIGURE 1:**
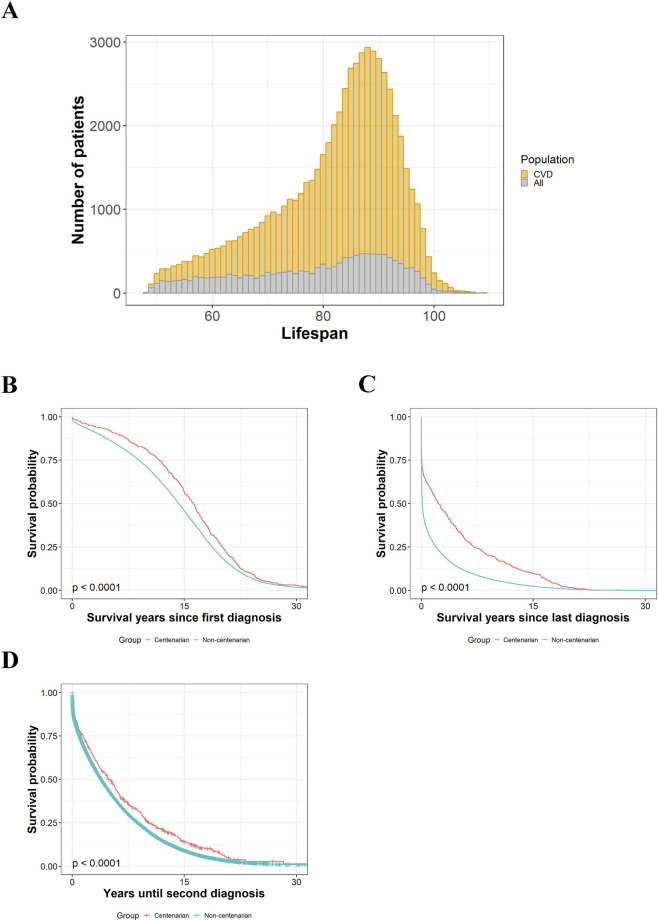
Analysis of survival in individuals with cardiovascular diseases. **(A)** Distribution of CVD patients (*n* = 50,003) vs. rest of the population (*n* = 13,399) as a function of age of decease. **(B)** Survival of centenarians (n = 501) vs. non-centenarians (n = 49,502) since the first diagnosis of CVD until death of individuals. Kaplan-Meier curves with the corresponding log-rank *p*-value. **(C)** Survival of centenarians (n = 501) vs. non-centenarians (n = 49,502) since the last diagnosis of CVD until death of individuals. Kaplan-Meier curves with the corresponding log-rank *p*-value. **(D)** Analysis of centenarians *(n =* 501) vs. non-centenarians *(n* = 49,502) since the first diagnosis of CVD until the second one. Kaplan-Meier curves with the corresponding log-rank *p*-value.

From the whole cohort, which comprised a total of 63,402 individuals deceased in Gipuzkoa between 2014 and 2022, 649 were centenarians. The first step was to explore the incidence of cardiovascular diseases in our two populations, and we saw that more than the 75% of individuals of both groups had at least one diagnosis of CVDs. In the case of centenarians, there were 501 (77.20%) of them, while in the non-centenarian group the incidence was of 78.88% with 49,502 individuals. The majority of individuals with CVD had a lifespan around 85–90 years, near the mean lifespan of the population (Figure 1A). Related to this, non-centenarians suffering from CVDs had a mean lifespan 4 years higher than of those free of disease, while in centenarians, lifespan of both groups was similar. The proportion of women and men with and without CVDs was similar in non-centenarians, but in centenarians, a slightly higher proportion of women had CVDs (Supplementary Table S2).

Moreover, we found significant differences in the incidence of some specific CVDs between the two groups. More precisely, non-centenarians displayed a higher incidence of ischemic heart diseases, diseases of arteries, arterioles and capillaries, and other forms of heart disease (all of them, *p* < 0.001). They also had more incidence of diseases of veins, lymphatic vessels and lymph nodes (*p* = 0.01). In contrast, the incidence percentages of the cerebrovascular disease and hypertensive disease groups were similar (Table 1).

**TABLE 1:** Incidence of different cardiovascular diseases in centenarians and non-centenarians.

Cardiovascular disease	Non-centenarians (n = 62,753)	Centenarians (n = 649)
Any cardiovascular disease	49,502 (78.88%)	501 (77.20%)
Cerebrovascular diseases	17,918 (28.55%)	185 (28.51%)
Diseases of veins, lymphatic vessels and lymph nodes	18,720 (29.83%)	164 (25.27%)
Hypertensive diseases	38,949 (62.07%)	411 (63.33%)
Ischemic heart diseases	11,171 (17.80%)	72 (11.09%)
Other forms of heart disease	31,959 (50.93%)	296 (45.61%)
Diseases of arteries, arterioles and capillaries	11,319 (18.04%)	54 (8.32%)

Number of individuals and percentages (%).

The next step was to characterize the impact of these diseases, and for that we focused on the records of diagnoses. Centenarians had their first CVD diagnosis more than 15 years later than non-centenarians. They also displayed a smaller proportion of multiple diagnoses, with 64.67% of them having more than three diagnoses and 54.09% having more than three different diagnoses, while in non-centenarians these numbers were higher (77.02% and 71.33%, respectively). As other conditions could also influence the effect of CVDs, we compared the prevalence of diabetes and obesity, observing that centenarians had notably lower incidence of both diseases by far. In addition, we explored differences in medications, seeing that centenarians had a lower mean number of prescribed cardiovascular drugs. We then focused on the specific case of antihypertensive drugs and, in this case, less than a 1% of the centenarian population had records of this prescription, with non-centenarians’ proportion being of 4%. Similarly, non-centenarians prescribed with lipids lowering drugs doubled centenarians. In line with the prevalence of diabetes, non-centenarians also needed more anti-diabetes drugs than centenarians (Table 2).

**TABLE 2:** Comparison of CVD diagnoses and treatments between centenarians and non-centenarians

Variable	CVD + non-centenarians (n = 49,502)	CVD + centenarians (n = 501)
Mean age of first diagnosis	68.88 ± 11.18	86.15 ± 7.43
Number of total diagnoses of cardiovascular diseases		
1	4,273 (8.63%)	60 (11.98%)
2	3,698 (7.47%)	65 (12.97%)
3	3,405 (6.88%)	52 (10.38%)
>3	38,126 (77.02%)	324 (64.67%)
Number of diagnoses of different cardiovascular diseases		
1	4,938 (9.98%)	71 (14.17%)
2	4,660 (9.41%)	84 (16.77%)
3	4,592 (9.28%)	75 (14.97%)
>3	35,312 (71.33%)	271 (54.09%)
Other related conditions		
Diabetes	18,649 (37.67%)	79 (15.77%)
Obesity	7,595 (15.34%)	13 (2.59%)
Pharmaceutic prescription		
At least 1 CVD drug	33,358 (67.39%)	354 (70.66%)
At least 1 antihypertensive drug	2,036 (4.11%)	4 (0.8%)
At least 1 lipid lowering drug	16,032 (32.39%)	83 (16.57%)
Mean number of CVD drugs	7.41 ± 6.37	6.10 ± 4.94
At least 1 diabetes drug	10,249 (20.70%)	33 (6.59%)

^a^ The information included total number of CVD diagnoses, number of different types of CVD diagnoses, number of individuals with conditions related to CVDs, and number of individuals with drugs prescribed. Number of individuals and percentages (%) are presented.

As we wanted to compare the repercussions of these CVDs in the survival of our two population groups, we built Kaplan-Meier curves. First, we assessed survival since the first diagnosis of CVD until death of individuals, seeing that while both curves displayed a similar trajectory, centenarians had extended survival (*p* < 0.001) (Figure 1B). Next, we studied survival since the last diagnosis of CVD, in this case observing a clear decay in non-centenarians’ curve, with more than 50% of them dying in the first year. On the other hand, centenarians’ curve extended through time displaying extended survival (*p* < 0.001) (Figure 1C). To complete this analysis, we studied recurrent diagnoses, evaluating years from the first CVD diagnosis until the second one. Similarly to the previous case, centenarians’ curve was fit over that of non-centenarians’, representing a more marked delay of posterior diagnoses (*p* < 0.001) (Figure 1D). We also evaluated survival since first diagnosis through a Cox regression, revealing that centenarians lived more than non-centenarians, even after adjusting by sex, age of first CVD diagnosis, the presence of diabetes and obesity, and drugs (Supplementary Figure 1).

**FIGURE 2:**
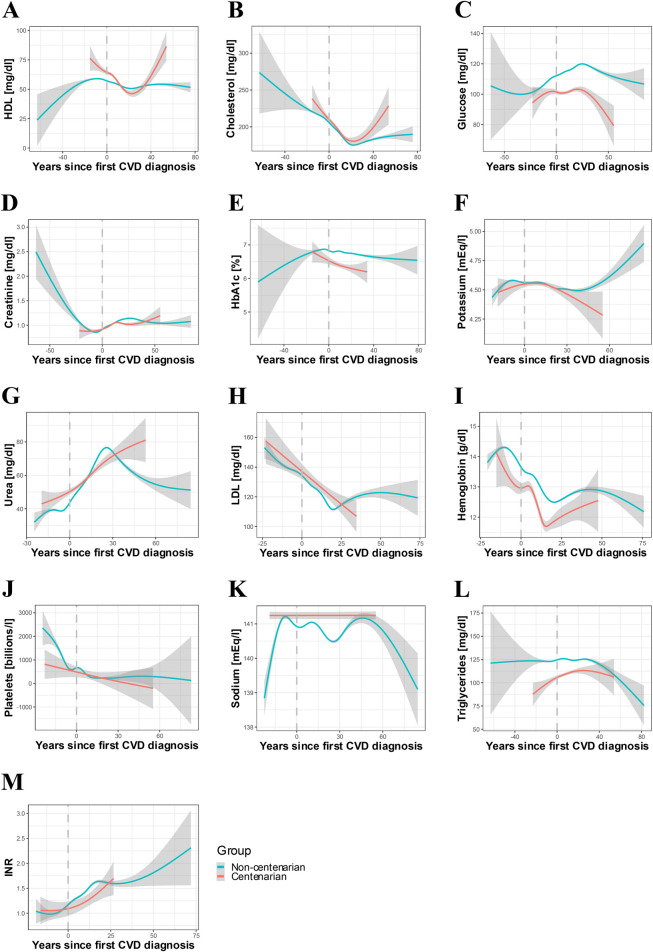
Trajectories of laboratory parameters in individuals with CVDs. Representation of the evolution of different laboratory variables as a function of the years relative to the first CVD diagnosis in centenarians *(n =* 501) vs. non-centenarians *(n* = 49,502). Dashed line represents year of first diagnosis. GAM models with CI (95%) **(A)** HDL **(B)** Total cholesterol **(C)** Glucose **(D)** Creatinine **(E)** Glycosylated hemoglobin **(F)** Potassium **(G)** Urea **(H)** LDL **(I)** Hemoglobin **(J)** Platelets **(K)** Sodium **(L)** Triglycerides **(M)** INR.

The last step was to evaluate the biological profiles in CVD context. For that, first we compared the results of the last blood analyses among both groups. We found significant differences in the levels of glucose, hemoglobin, glycosylated hemoglobin, INR, triglycerides, and sodium (Table 3), agreeing with previous studies of the group revealing differences among the two populations (Cruces-Salguero et al., 2022). Next, we studied differences in trajectories through NLMMs, finding significant differences in several variables (Table 4). First, at the moment of diagnosis, centenarians displayed higher levels of HDL, which decreased after CVD diagnosis and were later recovered. On the other hand, in non-centenarians, HDL suffered a mild decay after diagnosis and stayed stable afterwards (Figure 2A). The trajectory of cholesterol in centenarians was similar to that of HDL, while in non-centenarians it did not experiment such a clear recovery (Figure 2B). In the case of glucose, its levels exhibited a small increase after diagnosis followed by a decay in centenarians, whereas non-centenarians’ increase was more pronounced (Figure 2C). Similarly, creatinine displayed an increase in both groups but non-centenarians’ was more marked (Figure 2D). The levels of glycosylated hemoglobin were significantly higher in centenarians at the moment of diagnosis, and presented a decay in centenarians and remained stable in non-centenarians (Figure 2E). Regarding potassium, trajectories of centenarians and non-centenarians after CVD diagnosis were similar, although after 30 years it displayed a marked increase in non-centenarians and a decrease in centenarians, despite the CI being wide (Figure 2F). Finally, urea levels increased in both groups, exhibiting a sudden peak and decrease in non-centenarians and staying more stable in centenarians (Figure 2G). The rest of the variables did not display remarkable changes among both groups (Figures 2H–M). These results reveal significant differences in biological trajectories before and since diagnosis of CVDs.

**TABLE 3:** Comparison of CVD laboratory data between centenarians and non-centenarians**.**

Variable	Non-centenarians (n = 49,502)	Centenarians (n = 501)	*p*-value	FDR
*Total cholesterol (mg/dL)*	177.63 ± 46.21	179.64 ± 38.77	0.428	0.506
*HDL (mg/dL)*	51.21 ± 17.18	53.28 ± 18.07	0.082	0.153
*LDL (mg/dL)*	121.16 ± 33.69	118.91 ± 34.45	0.579	0.579
*Glucose (mg/dL)*	112.85 ± 43.26	100.29 ± 32.01	**< 0.001**	**< 0.001**
*Creatinine (mg/dL)*	1.10 ± 0.69	1.07 ± 0.47	0.172	0.249
*Hemoglobin (g/dL)*	12.61 ± 2.07	12.23 ± 1.81	**0.005**	**0.013**
*Glycosylated hemoglobin (%)*	6.24 ± 1.12	5.97 ± 0.81	**< 0.001**	**< 0.001**
*INR*	1.51 ± 1.62	1.14 ± 0.39	**< 0.001**	**< 0.001**
*Platelets (billions/L)*	233.98 ± 1,208.69	222.08 ± 99.82	0.100	0.162
*Potassium (mEq/L)*	4.55 ± 0.56	4.58 ± 0.58	0.222	0.289
*Sodium (mEq/L)*	140.80 ± 4.09	141.29 ± 4.12	**0.011**	**0.025**
*Triglycerides (mg/dL)*	115.29 ± 65.93	105.56 ± 46.34	**< 0.001**	**< 0.001**
*Urea (mg/dL)*	59.65 ± 39.02	60.83 ± 34.46	0.498	0.539

Data from the last blood analysis of individuals. Mean ± SD. The *p*-value of the Student’s t-test or Mann-Whitney *U* test (Shapiro-Wilk test *p* < 0.05) is reported, along with *p-*value after correcting for multiple comparisons (false discovery rate, FDR). Statistical significance is represented in bold (*p* < 0.05).

**TABLE 4:** Non-linear mixed models of the evolution of laboratory variables.

Variable	Intercept		Years to CVD diagnosis	Group		Years to CVD diagnosis: Group
​	Estimate	*p*-value	Min. *p*-value	Estimate	*p*-value	Min. *p*-value
*Total cholesterol (mg/dL)*	253.33	**< 0.001**	**< 0.001**	−79.74	0.529	**0.023**
*HDL (mg/dL)*	54.43	**< 0.001**	**< 0.001**	151.75	**< 0.001**	**< 0.001**
*LDL (mg/dL)*	154.43	**< 0.001**	**< 0.001**	19.73	0.457	0.213
*Glucose (mg/dL)*	40.29	**< 0.001**	**< 0.001**	44.98	0.354	**0.006**
*Creatinine (mg/dL)*	1.8	**< 0.001**	**< 0.001**	0.72	0.152	**0.03**
*Hemoglobin (g/dL)*	16.48	**< 0.001**	**< 0.001**	−0.76	0.295	0.233
*Glycosylated hemoglobin (%)*	5.77	**< 0.001**	**< 0.001**	10.22	**0.034**	**0.026**
*INR*	0.88	**< 0.001**	**< 0.001**	0.24	0.785	0.542
*Platelets (billions/L)*	2,089.4	**< 0.001**	**< 0.001**	−2,208.4	0.234	0.186
*Potassium (mEq/L)*	4.6	**< 0.001**	**< 0.001**	−0.23	0.096	**0.006**
*Sodium (mEq/L)*	141.5	**< 0.001**	**< 0.001**	−0.058	0.949	0.595
*Triglycerides (mg/dL)*	125.33	**< 0.001**	**< 0.001**	−156.22	0.157	**0.069**
*Urea (mg/dL)*	24.22	**< 0.001**	**< 0.001**	2.37	0.8	**0.027**

Results from NLMMs, including splines for time (years to CVD diagnosis) and their interaction with group (centenarian (n = 501) vs. non-centenarian (n = 49,502)). The intercept represents the estimated mean value of the variable at the first measurement in the reference group (non-centenarians). “Years to CVD diagnosis” *p*-values refer to the overall time-related change in the reference group. “Group” and its *p*-value represent differences between groups at the first measurement. Interaction *p*-values (Years to CVD diagnosis: Group) indicate whether the temporal trajectories differ between groups. The minimum *p*-value among spline terms is reported for the time- and interaction-related effects as an approximate indicator of significance. Statistical significance is represented in bold (*p* < 0.05).

## Discussion

CVDs represent the main cause of death worldwide; however, their effect on centenarians is still vaguely explored. Until now, evidence suggests that centenarians may exhibit a unique cardiovascular profile, potentially delaying both the onset and progression of disease (Galioto et al., 2008; Summer et al., 2025). Consistent with this, our results showed an almost 20-year delay in the onset of the first CVD in centenarians compared to non-centenarians. Regarding incidence, we found similar overall rates between centenarians and non-centenarians, but centenarians did have significantly lower incidence of some of the most lethal conditions, such as ischemic heart disease. These findings are in line with studies reporting reduced incidences of CVDs, such as myocardial infarction, in centenarians (Galioto et al., 2008). These benefits have been attributed to a combination of protective genetic factors and healthy lifestyle, including diet, reduced stress levels, and physical activity (Galioto et al., 2008; Summer et al., 2025; Puca et al., 2018; Dai et al., 2024), which leads to a healthier vasculature system (Buscemi et al., 2024; Jachymczyk et al., 2006/01).

In the specific case of hypertension, in our cohort the incidence was high, exceeding 50% in both centenarians and non-centenarians. There is controversy in literature, with some studies reporting lower incidence in centenarians (Galioto et al., 2008) and others suggesting the opposite (Zyczkowska et al., 2006). In particular, in the Danish Longitudinal Centenarian Study, the prevalence of hypertension in centenarians was similar to ours (Andersen et al., 2006). It is important to highlight that despite the high incidence of hypertension and overall CVDs in centenarians, we still found that they presented fewer diagnoses and prescribed drugs, along with related pathologies such as diabetes. In addition, the survival analysis followed the same trend, with centenarians displaying extended survival both since the first and last CVD diagnosis. These results might suggest a potential resilience to CVDs in this population group, despite the prevalence of some conditions such as hypertension.

Centenarians have been reported to present “healthier” biological profiles, exhibiting improved lipid metabolism (He et al., 2016), which may contribute to a relative protection against CVD. We analyzed trajectories of different biological parameters recorded in the EHRs, and found that both HDL and total cholesterol levels in centenarians described a U-shape trajectory, with a decay after the first CVD diagnosis followed by a subsequent increase. While low HDL and high LDL or total cholesterol are traditionally associated with increased CVD risk (Duncan et al., 2019), studies specifically examining lipid trajectories in the context of CVD are limited. For instance, the Framingham study found that unfavorable HDL trajectories, related to low and decreasing levels, were linked to higher risk of atherosclerotic cardiovascular disease (ASCVD) mortality (Duncan et al., 2019). In our cohort, HDL levels decreased in both centenarians and non-centenarians around CVD diagnosis, but centenarians demonstrated a faster recovery of favorable levels. Total cholesterol showed a similar pattern, suggesting that the post-diagnosis increase was largely driven by HDL rather than LDL, and therefore may not reflect an adverse lipid profile.

Regarding glucose metabolism, a previous study associated increasing triglyceride-glucose index with CVD mortality (Lee et al., 2024/10). We found a modest post-diagnosis increase in glucose levels in centenarians, followed by a decline, whereas non-centenarians exhibited a marked glucose peak after CVD diagnosis, indicating a more hazardous glycemic profile. In relation to this, high glycosylated hemoglobin levels have been associated with cardiovascular events in patients with multivessel coronary artery disease and diabetes (Rezende et al., 2020). In our study, and consistent with glucose results, centenarians showed a decay in glycosylated hemoglobin levels after CVD diagnosis, while in non-centenarians remained more stable. Even though further and more specific research on this topic should be conducted, our results might indicate that centenarians’ resilience against CVDs is in part due to a protector glycemic profile.

Besides, both centenarians and non-centenarians displayed a creatinine increase after CVD diagnosis; however, in non-centenarians this increase extended continuously over more time, while in centenarians there was a decay, and the last increase exhibited a wide CI due to the limited sample size. As increased creatinine levels have also been linked to higher risk of developing CVDs (Zhai et al., 2023; Cho et al., 2024), these results may suggest a greater vulnerability in non-centenarians.

Additionally, two studies found an association between abnormal potassium levels and cardiovascular mortality (Fan et al., 2024; Cheungpasitporn et al., 2017). In our cohort, non-centenarians experienced a sudden increase in potassium approximately 50 years after CVD diagnosis, potentially reflecting extreme risk cases. Finally, regarding urea, it has been described that elevated levels increased the risk of both CVD (Liu et al., 2024; Lan et al., 2021) and cardiovascular mortality (Shen et al., 2024), which fit our findings of non-centenarians’ trajectory rising right after CVD diagnosis and centenarians’ increase being notably slower. All in all, the comparison of these trajectories might suggest that, despite centenarians still suffering the biological consequences of CVDs, these effects are generally more moderate and followed by faster recovery compared to non-centenarians. However, further and more specific analyses should be done to confirm these results, since the heterogeneity of CVDs and the limited sample size in centenarians could be distorting the results.

Additional limitations of this study include selection bias, derived from the analysis of only deceased individuals rather than the general population, which limits the interpretation of incidence and survival patterns. Survivor bias is also inherent to the study of long-lived individuals. The sample size across groups was also highly imbalanced, which could affect statistical power. Differences in the use of healthcare resources, particularly among centenarians, could also influence the number of recorded diagnoses in EHRs; however, survival and biological patterns still hinted at potential resilience in centenarians. Finally, although diabetes and obesity were taken into account, residual confounding derived from other comorbidities could distort the results.

In summary, our findings demonstrate a resilience of CVDs in centenarians, with fewer severe diseases, extended survival, and favorable biological trajectories.

## Data Availability

The original contributions presented in the study are included in the article/Supplementary Material, further inquiries can be directed to the corresponding author.
