# Supplementary material for: Electronic health records reveals resilience patterns of cardiovascular disease in Basque centenarians
Source: Front Aging. 2026 Mar 31;7:1805053. doi: 10.3389/fragi.2026.1805053 (PMC13076568; doi:10.3389/fragi.2026.1805053)
Supplement: Supplementary file 1 [file Supplementaryfile1.docx]

**Supplementary File S1**

**Supplementary Table 1. List of ICD-9 and ATC codes included in the analysis**

**Supplementary Table 2. Demographic characteristics of the study cohort**

**Supplementary Figure 1. Multivariate Cox regression of CVD patients.**

**Supplementary Table 1. List of ICD-9 and ATC codes included in the analysis.**

| **ICD-9/ATC codes** |  |
| --- | --- |
| **CVD diagnoses** | |
| 401-405 | Hypertensive diseases |
| 410, 411.[0, 1, 8], 412-414, 429.2 | Ischaemic heart diseases |
| 420.9, 421.[0, 9], 422.9, 423,  424.[0-3, 0, 9], 425.[0-6,9], 426-428, 429.[0, 1, 3,5-9] | Other forms of heart disease |
| 046.3, 348.39, 430-434, 435.9, 436, 437.[0-6,8-9], 438, 443.29 | Cerebrovascular diseases |
| 440-442, 443.[0-28,82-9], 444, 445, 447, 448 | Diseases of arteries, arterioles and capillaries |
| 289.[1-3], 451-454, 456, 457.[1, 2, 8, 9], 459.[1-81,9] | Diseases of veins, lymphatic vessels and lymph nodes, not elsewhere classified |
| **Related conditions** | |
| 250, 648.0, 790.29 | Diabetes |
| 278.0 | Obesity |
| **Drugs** | |
| C | Cardiovascular system drugs |
| C02 | Antihypertensives |
| C10 | Lipid lowering drugs |
| A10 | Anti-diabetes drugs |

|  | **Non-centenarians**  **(n = 62,753)** | | **Centenarians**  **(n = 649)** | |
| --- | --- | --- | --- | --- |
|  | **CVD+**  **(n = 49,502)** | **CVD-**  **(n = 13,251)** | **CVD+**  **(n = 501)** | **CVD-**  **(n = 148)** |
| Age | 82.83 ± 10.53 | 78.89 ± 13.25 | 101.91 ± 1.77 | 102.6 ± 2.2 |
| Sex | 24,566 women  (49.63%)  24,936 men  (50.37%) | 6,354 women  (47.95%)  6,897 men  (52.05%) | 436 women  (87.03%)  65 men  (12.97%) | 121 women  (81.76%)  27 men  (18.24%) |
| Nursing home | 7,315 yes (14.78%)  42,187 no (85.22%) | 1,645 yes (12.41%)  11,606 no (87.59%) | 145 yes (28.94%)  356 no (71.06%) | 42 yes (28.38%)  106 no (71.62%) |

**Supplementary Table 2. Demographic characteristics of the study cohort.**

**
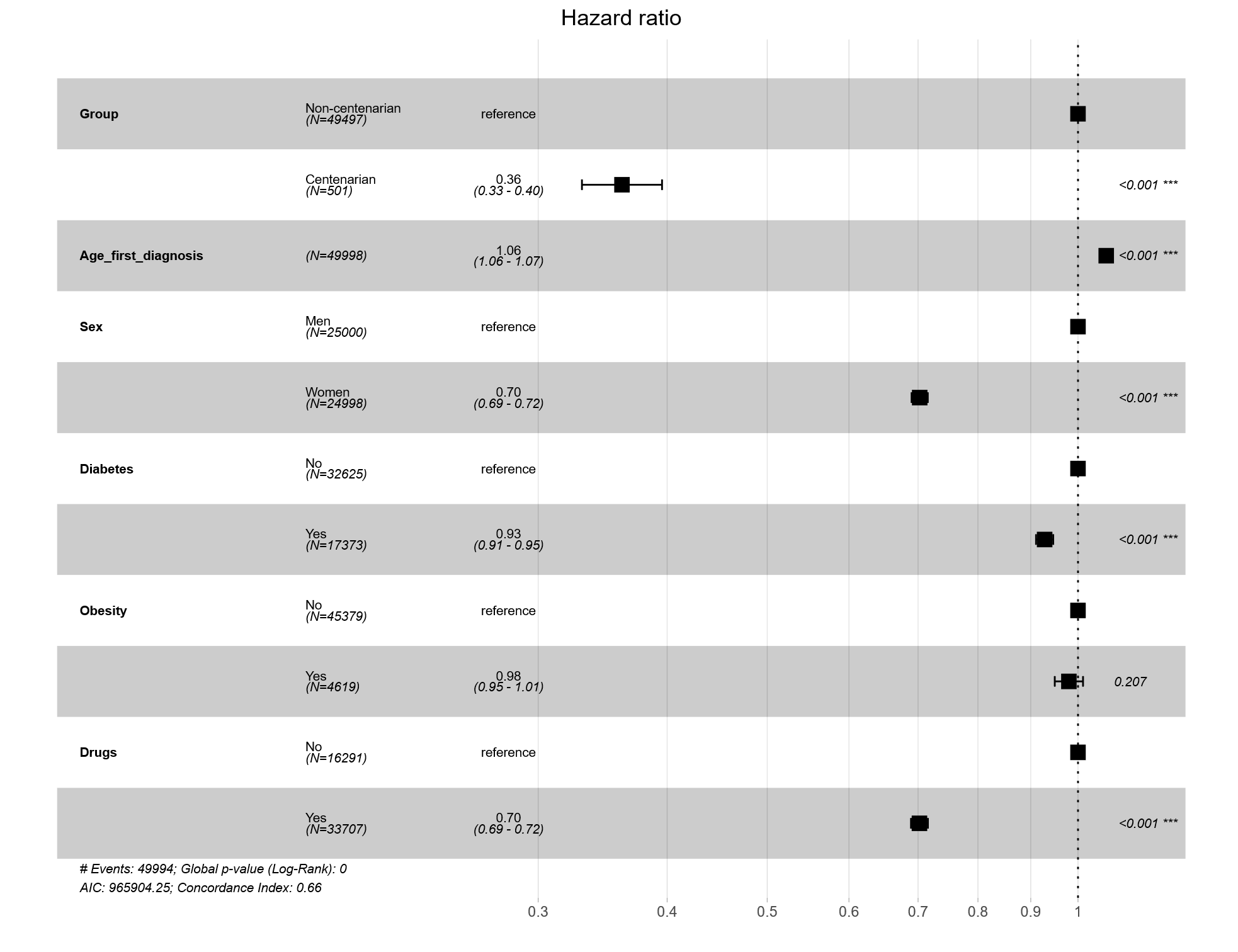
**

**Supplementary Figure 1. Multivariate Cox regression of CVD patients.** Survival time was calculated since the first recorded CVD diagnosis until death of individuals. The model includes population group (centenarian or non-centenarian), age at diagnosis, sex, diabetes, obesity, and pharmacological treatment as covariates. Hazard ratios (HRs) and the corresponding *p-*values are shown.
